# Thermoresponsive Molecular Brushes with a Rigid-Chain Aromatic Polyester Backbone and Poly-2-alkyl-2-oxazoline Side Chains

**DOI:** 10.3390/ijms222212265

**Published:** 2021-11-12

**Authors:** Elena Tarabukina, Emil Fatullaev, Anna Krasova, Maria Sokolova, Mikhail Kurlykin, Igor Neelov, Andrey Tenkovtsev, Alexander Filippov

**Affiliations:** 1 Institute of Macromolecular Compounds, Russian Academy of Sciences, 199004 Saint Petersburg, Russia; len.ta@mail.ru (E.T.); krasova_anna@bk.ru (A.K.); pmarip@mail.ru (M.S.); mike_x@mail.ru (M.K.); tenkovtsev@yandex.ru (A.T.); afil@imc.macro.ru (A.F.); 2 St. Petersburg National Research University of Information Technologies, Mechanics and Optics (ITMO University), 197101 Saint Petersburg, Russia; ximik53@yandex.ru

**Keywords:** synthesis, molecular brushes, poly-2-alkyl-2-oxazolines, solutions, structural and conformation characteristics, thermoresponsiveness, self-assembly

## Abstract

A new polycondensation aromatic rigid-chain polyester macroinitiator was synthesized and used to graft linear poly-2-ethyl-2-oxazoline as well as poly-2-isopropyl-2-oxazoline by cationic polymerization. The prepared copolymers and the macroinitiator were characterized by NMR, GPC, AFM, turbidimetry, static, and dynamic light scattering. The molar masses of the polyester main chain and the grafted copolymers with poly-2-ethyl-2-oxazoline and poly-2-isopropyl-2-oxazoline side chains were 26,500, 208,000, and 67,900, respectively. The molar masses of the side chains of poly-2-ethyl-2-oxazoline and poly-2-isopropyl-2-oxazoline and their grafting densities were 7400 and 3400 and 0.53 and 0.27, respectively. In chloroform, the copolymers conformation can be considered as a cylinder wormlike chain, the diameter of which depends on the side chain length. In water at low temperatures, the macromolecules of the poly-2-ethyl-2-oxazoline copolymer assume a wormlike conformation because their backbones are well shielded by side chains, whereas the copolymer with short side chains and low grafting density strongly aggregates, which was visualized by AFM. The phase separation temperatures of the copolymers were lower than those of linear analogs of the side chains and decreased with the concentration for both samples. The LCST were estimated to be around 45 °C for the poly-2-ethyl-2-oxazoline graft copolymer, and below 20 °C for the poly-2-isopropyl-2-oxazoline graft copolymer.

## 1. Introduction

The synthesis of rigid-chain polymers in the middle of the 20th century was a breakthrough in polymer science and technology, which made it possible to obtain new ultra-strong materials. Rigid-chain polymers usually include polymers with a Kuhn segment length A of more than 10 nm. The high rigidity of macromolecules predetermines their elongated state in the direction of the main chain and often provides lyotropic mesomorphism [1,2,3,4,5]. Such features are useful for creating high-strength fibers and films and self-reinforcing plastics, including those with specific optical and conductive properties [6,7,8,9].

The establishment of the correlation of molecular characteristics of such polymers with their structure has been and is still being actively pursued by many scientific groups. Analysis of the various molecular structures showed that the high chain equilibrium and kinetic rigidity is the effect of a specific chemical structure of macromolecules [10,11]. The main reason for the increase in A of chain molecules is the presence in their repeating units of structural elements that prevent rotation around the chain direction, such as double bonds, cyclic fragments, and a ladder structure [12,13,14,15]. An increase in equilibrium rigidity is facilitated by chain conjugation, such as, for example, in polyisocyanates, and specific non-covalent interactions, such as in DNA and polysaccharides [16,17,18,19,20,21]. In macromolecules with a comb-like structure, increased rigidity is induced by steric obstacles arising between the side chains, which impede the bending and folding of the main chain as a result of intramolecular thermal movement of the units [11,22,23].

Research on comb-like polymers, or more precisely, molecular brushes, intensified at the end of the 20th century, when new synthetic approaches were developed that allowed the controlled synthesis of high-molecular compounds of various architectures [24,25,26,27,28,29]. As a result, the main regularities of the behavior of graft copolymers in solutions were established; in particular, the influence of the size of the main and side chains, as well as the grafting density of the latter, on the hydrodynamic characteristics and conformation of comb-like macromolecules was revealed [23,30,31,32,33,34,35,36,37,38,39]. In the formation of properties in solutions of amphiphilic comb-shaped macromolecules, in which the chemical nature of the main and side chains is very different, the affinity to the solvent of various blocks of the graft copolymer is of great importance [40,41,42,43,44,45]. Accordingly, by varying the chemical structure of the main and side chains, their sizes, and grafting density, it is possible to control the self-organization of molecular brushes in selective solvents. For example, molecules of graft copolymers with a flexible main chain of polyimides and side chains of polymethyl methacrylate in selective solvents are capable of assuming an extended or compact conformation depending on the grafting density of side chains and their length, as well as the hydrodynamic quality of the solvent with respect to the components [46,47,48]. A significant role in the formation of the properties of molecular brushes in solutions is played by the rigidity of their structural elements: the main and side chains [11,38,49,50]. It has been shown that the presence of rigid-chain fragments in the side chains can lead to their orientational order in solution. On the other hand, grafting long flexible chains onto a rigid aromatic backbone prevents aggregation when grafting is sufficiently dense.

Recently, special attention has been attracted by amphiphilic stimuli-sensitive graft copolymers, solutions of which are characterized by a nonlinear response to a weak external effect, for example, to a change in the temperature or acidity of the medium, to irradiation with light at a certain wavelength, etc. [51,52,53,54,55,56,57]. Typically, such brushes are built with a hydrophobic or less commonly hydrophilic backbone and water-soluble stimulus-sensitive side chains. Thermoresponsive poly-2-alkyl-2-oxazolines (PAlOx) are promising as grafted blocks [58,59,60,61,62]. Biocompatible linear PAlOx have good prospects for use in medicine and biotechnology; the conditions of controlled synthesis have been reliably established for them and the basic correlations of behavior in solutions have been revealed [63,64,65,66,67]. In particular, it was shown that the dehydration temperature of PAlOx units decreases with an increase in the size of the side radical in their chains [68]. Accordingly, the lower critical dissolution temperature (LCST) for poly(2-ethyl-2-oxazoline) (PEtOx) solutions is about 30 °C higher than the LCST for poly-2-isopropyl-2-oxazoline (PiPrOx). This pattern is retained for graft copolymers in which aromatic polyesters served as the main chain and PEtOx or PiPrOx as side chains [69,70,71]. In this case, the nature of self-organization, namely, compaction or aggregation, in solutions of the discussed molecular brushes depended on the grafting density of side chains.

**Figure 1:**
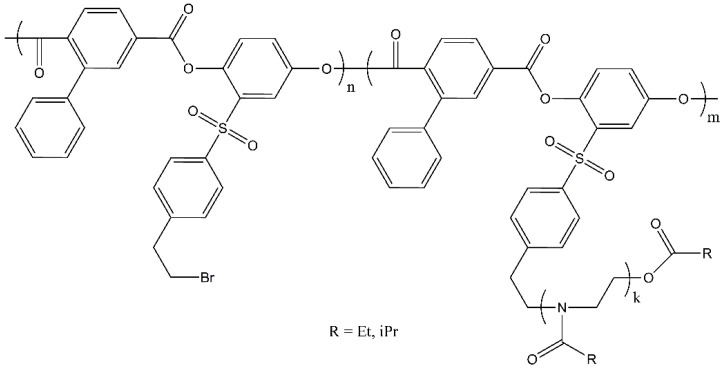
Chemical structure of the APE_r.ch._-graft-PEtOx and APE_r.ch._-graft-PiPrOx graft copolymers.

All the cited works investigated graft copolymers in which flexible-chain polymers were used as the main chain. It seems interesting to analyze how the behavior of molecular brushes with PAlOx side chains will change if the backbone is a rigid chain polymer. In connection with the above, the objectives of this work are: (i) the development of approaches to the controlled synthesis of copolymers (APE_r.ch._-graft-PAlOx), in which thermosensitive PEtOx or PiPrOx are grafted to a rigid-chain aromatic polyester (APE_r.ch._); (ii) determination of hydrodynamic and conformational characteristics of the synthesized APE_r.ch._-graft-PAlOx; and (iii) the study of the thermal response of aqueous solutions of the synthesized molecular brushes and its dependence on the chemical structure of the side PAlOx chains. The structure of APE_r.ch._-graft-PAlOx is shown in Figure 1.

## 2. Results and Discussion

### 2.1. Synthesis 

#### 2.1.1. Synthesis of the Polyester Multifunctional Macroinitiator

2-(4-(2-bromoethyl)phenylsulfonylhydroquinone was chosen as a polycondensation monomer containing functional groups suitable for initiating cationic polymerization of 2-alkyl-2-oxazolines. The choice of this compound was determined by the fact that previously described [72] soluble alkylene-aromatic polyesters based on phenylsulfonylhydroquinone can be synthesized using the method of non-acceptor polycondensation. It is necessary to keep in mind that the presence of a 2-bromoethyl group sensitive to tertiary amines and alkali in the target monomer makes it impossible to apply traditional acceptor methods for polyester synthesis. On the other hand, it can be assumed that the direct nucleophilic substitution of bromine in the 2-bromoethyl group as a result of the attack of a phenolic hydroxyl in the absence of bases is an unlikely process.

**Figure 2:**
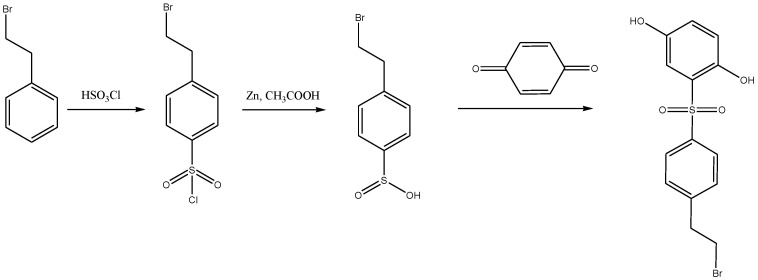
Synthesis of the functionalized hydroquinone comonomer.

Synthesis of 2-(4-(2-bromoethyl)phenylsulfonylhydroquinone was carried out using the previously published procedure [73], which includes the reaction of 2-phenylethyl bromide with chlorosulfonic acid, reduction of the corresponding sulfonyl chloride to sulfinic acid, and addition of the latter, under the conditions of the Michael reaction, to 1,4-benzoquinone (Figure 2).

**Figure 3:**
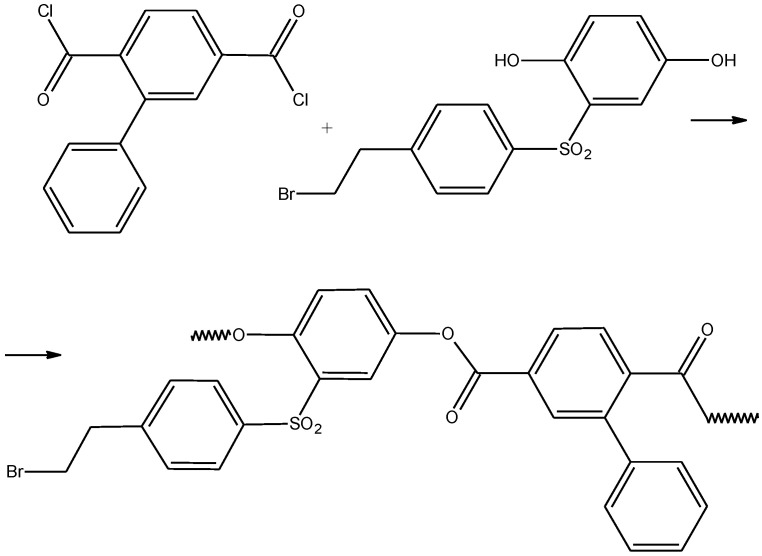
Synthesis of the APE_r.ch._ macroinitiator.

[1,1’-biphenyl]-2,5-dicarbonyl dichloride was used as a comonomer. The polymer synthesis was carried out under the conditions of acceptorless high-temperature polycondensation (Figure 3), which has proven itself well in the synthesis of aromatic polyesters [74]. 1-Chloro-naphthalene was used as a solvent. It was found that the optimal conditions for polycondensation are as follows: temperature of 200 °C, monomer concentration of 25 wt%, and reaction time of 2 h.

#### 2.1.2. Synthesis of the APE_r.ch._-Graft-PAlOx Copolymers with PEtOx or PiPrOx Side Chains

**Figure 4:**
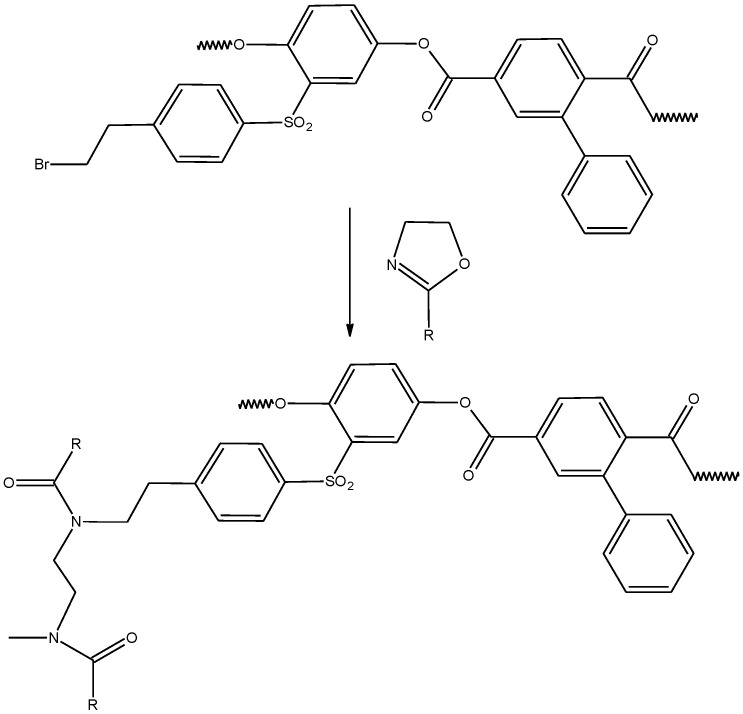
Synthesis of the APE_r.ch._-graft-PAlOx copolymers.

It is well known that aromatic polyesters are soluble in a limited number of solvents (chlorinated hydrocarbons, CF_3_COOH, m-cresol), which significantly limits the choice of a medium for cationic polymerization. It is obvious that protonic acids are unsuitable for this purpose, while chlorinated hydrocarbons are close to theta-solvents for synthesized macroinitiators at room temperatures [75]. It is well known that carrying out polymer analogous transformations in poor solvents cannot provide a sufficient degree of grafting due to the unavailability of a significant part of the functional groups. Usually, the thermodynamic quality of the solvent improves with increasing temperature. In this regard, the grafting of polyoxazoline chains (Figure 4) was carried out in tetrachloroethane at 150 °C.

**Figure 5:**
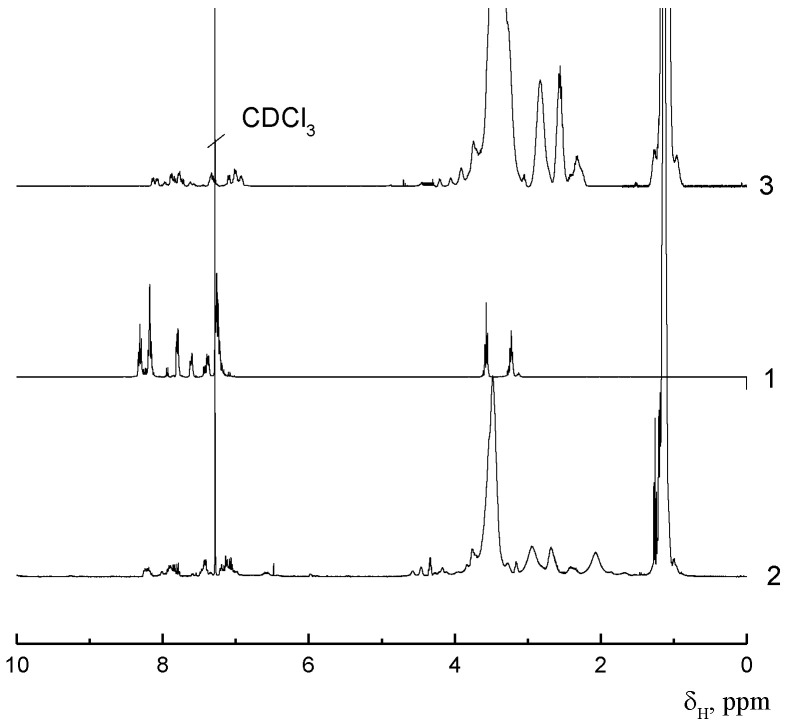
^1^H NMR spectra of the macroinitiator APE_r.ch._ (1), the APE_r.ch._-graft-PiPrOx (2), and the APE_r.ch._-graft-PEtOx (3).

**Figure 6:**
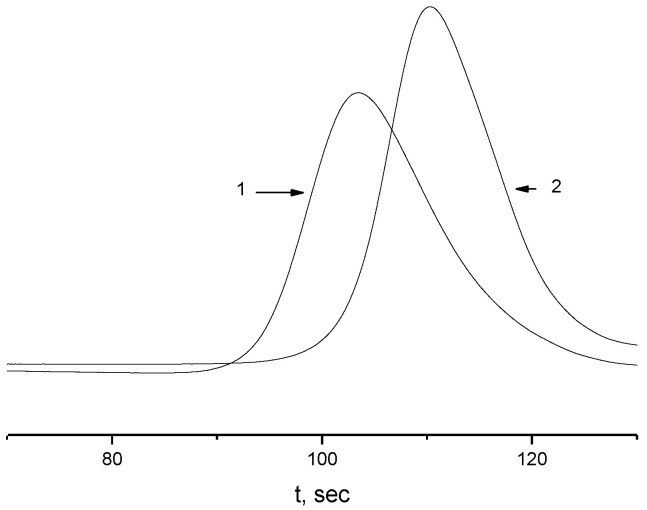
GPC traces of the APE_r.ch._-graft-PEtOx (1) and the APE_r.ch._-graft-PiPrOx (2).

**Figure 7:**
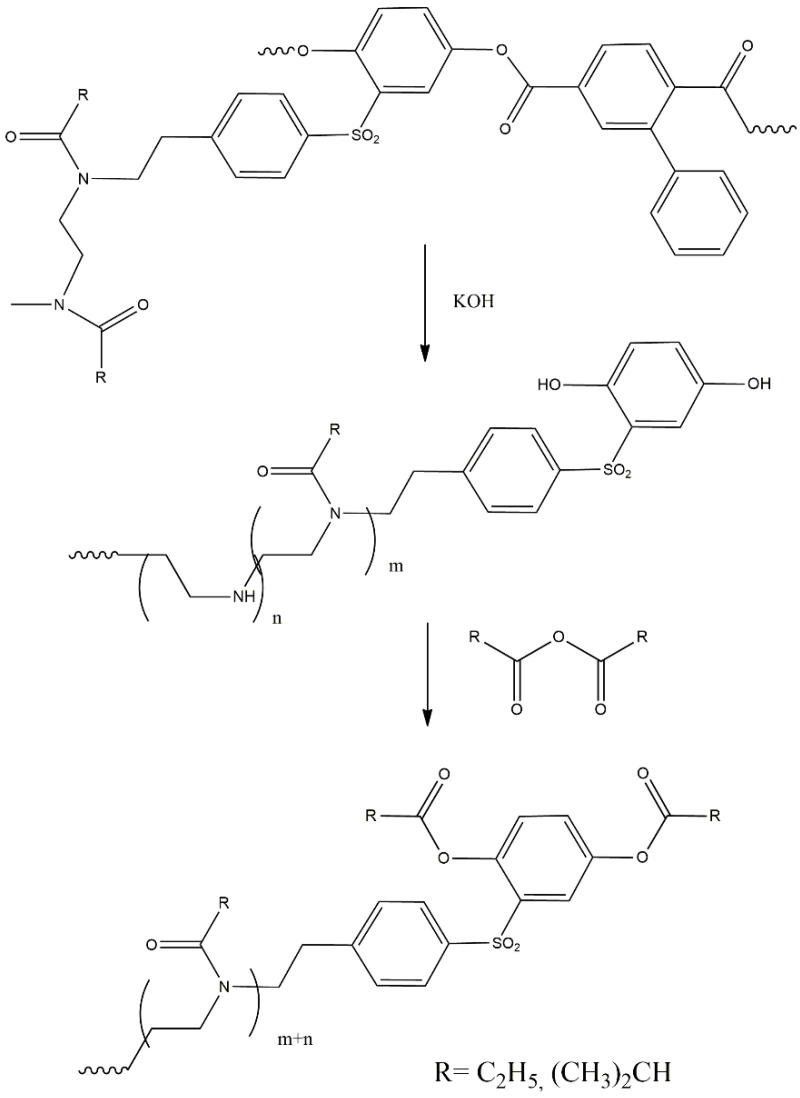
Selective destruction of synthesized graft-copolymers.

A comparison, for example, of the ^1^H NMR spectra of the macroinitiator and the grafted copolymer APE_r.ch._-graft-PEtOx shows (Figure 5) that both signals of aromatic protons of the main chain and signals related to the PEtOx side chains are present in the spectrum of the copolymer. Together with the monomodality of the obtained polymer, these data allow us to assert that the sample under investigation is a graft copolymer. It should be noted, however, the asymmetric shape of the GPC trace of the graft copolymer (Figure 6), which probably indicates some unevenness in the distribution of the side chains along the main one. In order to determine the molecular weight characteristics of the grafted chains and their grafting density, the main chains of copolymers were destroyed by alkaline hydrolysis under conditions that provide quantitative cleavage of ester groups [76] (Figure 7).

**Figure 8:**
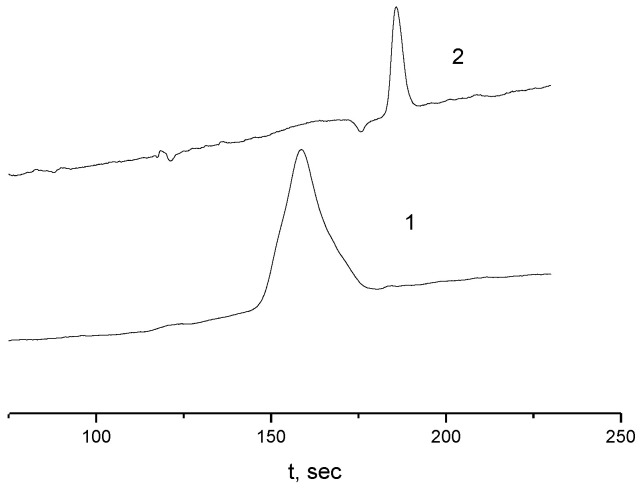
GPC traces of the PEtOx (1) and PiPrOx (2) isolated side chains.

Isolated side chains, after acylation with propionic anhydride (in the analysis of the APE_r.ch._-graft-PEtOx) or isobutyric anhydride (in the analysis of the APE_r.ch._-graft-PiPrOx), according to chromatographic data, had weight-average molar mass *M*_w_ = 7400 (polydispersity *M*_w_/*M*_n_ = 1.34) for APE_r.ch._-graft-PEtOx and *M*_w_ = 3400 (*M*_w_/*M*_n_ = 1.27) for APE_r.ch._-graft-PiPrOx (Figure 8). 

### 2.2. Molecular Characteristics and Equilibrium Rigidity of the APE_r.ch._ Macroinitiator and the Grafted Copolymers APE_r.ch._-graft-PEtOx and APE_r.ch._-graft-PiPrOx 

The molar masses and hydrodynamic characteristics of the macroinitiator and the graft copolymers are given in Table 1. First of all, we note that for the APE_r.ch._ in tetrachloroethane, a very low value of the second virial coefficient A_2_ was obtained. Consequently, tetrachloroethane is close in thermodynamic quality to the θ-solvent. This is also indicated by the absence of the concentration dependence of the hydrodynamic radius *R*_h_ (Figure S1), which is typical for θ-solvents in which thermodynamic interactions are absent. This fact makes it possible to roughly estimate the APE_r.ch._ equilibrium rigidity by the value of *R*_h_, using the relation valid for Gaussian chains:

*f* = 6^1/2^η_0_*P R*_g_, (1)

where *f* and *R*_g_ are the translational friction coefficient and the radius of gyration of macromolecules, respectively, and the invariant *P* is *P* = 5.1 [11]. This relationship is similar to the Flory–Fox equation for intrinsic viscosity. According to the Stokes equation used to calculate the hydrodynamic radius:

*f* = 6πη_0_*R*_h_, (2)

and therefore:

<*h*^2^>^1/2^ = 6π*R*_h_/*P*, (3)

where <*h*^2^>^1/2^ is the root-mean-square distance between the ends of the polymer chain, which, according to the Kuhn equation, is related to the contour length of the macromolecule *L* and the Kuhn segment length *A*:

<*h*^2^> = *LA*. (4)

The molar mass *M*_0-APE_ of the APE_r.ch._ monomer unit is 563 g·mol^−1^. Therefore, the macroinitiator molecule consists of *N*_APE_ = (*n* + *m*) = *M*_w_/*M*_0-APE_ = 26,500/563 = 47 monomer units, n and m being unsubstituted and substituted ones, respectively. The length of the macroinitiator monomer unit λ_0-APE_ can be estimated as the sum of the lengths of its bonds along the chain direction. Assuming that the length of all valence bonds of the main chain is close to 0.14 nm, and the valence angles are tetrahedral, in accordance with the APE_r.ch._ structural formula (Figure 1), we obtain λ_0-APE_ = 1.51 nm. Accordingly, the APE_r.ch._ contour length is *L*_APE_ = 71 nm. Substitution of the *R*_h_ and *L*_APE_ values into Equations (3) and (4) results in the Kuhn segment length *A* = 28 nm.

We emphasize once again that the approach used is a rough estimation of the APE_r.ch._ equilibrium rigidity. Indeed, the APE_r.ch._ macromolecule chain consists of only 71/28 = 2.6 Kuhn segments, and its behavior does not obey Gaussian statistics. However, the result obtained is important from the point of view, which allows us to reliably state that APE_r.ch._ is a typical rigid-chain polymer. Therefore, its conformation should be analyzed within the framework of the Porod model, that is, a worm-like chain with constant curvature. Several approaches are used to describe the hydrodynamic behavior of rigid-chain polymers (see [11]), for example, the model of a worm-shaped spherocylinder proposed by Yamakawa [77,78,79]. In any case, in order to quantitatively determine the equilibrium rigidity of a polymer from hydrodynamic data, it is necessary to study the homologous series.

**Figure 9:**
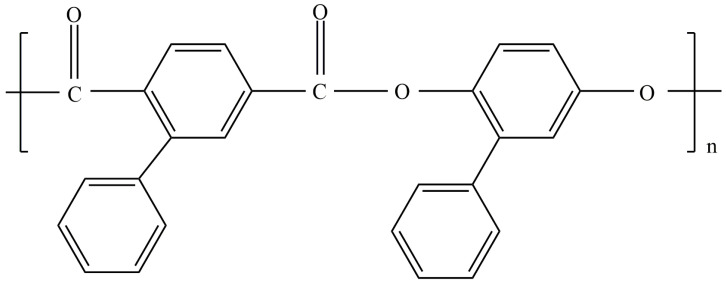
Chemical structure of the pAPE [80].

The long Kuhn segment for the APE_r.ch._ was expected, since its chain is built of alternating planar ester groups and para-phenylene rings. Such structures are characterized by the so-called “crankshaft” conformation [11], which provides high equilibrium rigidity. For example, the Kuhn segment length of a para-aromatic polyester (pAPE) (Figure 9) is 26 nm according to hydrodynamic data [80] and 20 nm according to flow birefringence data [81]. As seen in Figure 1 and Figure 9, the APE_r.ch._ and the pAPE differ very slightly (the structure of the main chains is identical); therefore, these polymers should have similar rigidity.

**Figure 10:**
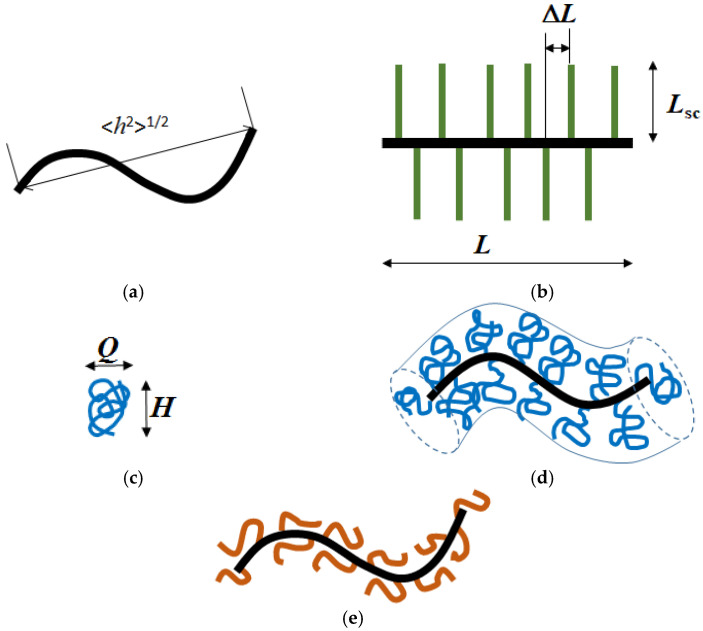
Model representation of the APE_r.ch._ (**a**), the APE_r.ch._-graft-PEtOx (**d**), and the APE_r.ch._-graft-PiPrOx (**e**) macromolecules; structural parameters of the chain *L*, **Δ***L*, and *L*_sc_ (**b**); and the Gaussian coil dimensions determination (**c**).

Thus, it can be argued with high probability that the APE_r.ch_. Kuhn segment length is within the range from 20 to 26 nm. Accordingly, the macroinitiator molecules have a curved thin rod conformation (Figure 10a). As a measure of the curving of a chain macromolecule, the ratio <*h*^2^>^1/2^/*L* can be considered, which varies from 1 for a straight rod to 0 for an infinitely long Gaussian chain. The value of <*h*^2^> can be estimated using the Porod equation for persistent chains [82,83]:

(5) $$\frac{<h^{2}>}{AL}=1-\frac{1-e^{-2L/A}}{2L/A}.$$

Therefore, the ratio <*h*^2^>^1/2^/*L* is 0.49 at *A* = 20 nm and 0.56 at *A* = 28 nm, that is, the length of the APE_r.ch._ macromolecule about twice exceeds the distance between the ends of its chain. As for the thickness *d* of the macroinitiator molecules, based on its structure, it is close to 2 nm, and the ratio *d*/*A* = (0.07–0.10) is typical for thin rigid-chain molecules.

To determine the conformation of the molecules of grafted copolymers, it is necessary to know a number of structural parameters, first of all, the length of the side chains *L*_sc_ (Figure 10b) and the grafting density *z* of the latter. It is easy to show that the value of *z* can be calculated from the relation:

(6) $$z=\frac{m}{n+m}=\frac{m}{N_{\mathrm{APE}}}=\frac{M_{\mathrm{cop}}-M_{\mathrm{APE}}}{N_{\mathrm{APE}}(M_{\mathrm{sc}}-M_{\mathrm{Br}})},$$

where *M*_cop_, *M*_APE_, *M*_sc_, *M*_Br_ = 79.9 g·mol^−1^ are the molar masses of the graft copolymer, the macroinitiator, the side chains, and bromine, respectively. According to NMR spectroscopy, the degrees of polymerization of the side chains *N*_sc_ were 75 and 30 for the APE_r.ch._-graft-PEtOx and APE_r.ch._-graft-PiPrOx (Table 2). Taking into account that the molar masses M_0-sc_ of the monomeric units of the side chains of PEtOx and PiPrOx are equal to 99 and 113 g·mol^−1^, it is easy to obtain the values M_sc_ = M_0-sc_·N_sc_ for the studied copolymers (Table 2).

Calculations using Equation (6) show that the grafting density of the APE_r.ch._-graft-PEtOx and the APE_r.ch._-graft-PiPrOx side chains differs by a factor of two (Table 2). Note that the *z* value for the APE_r.ch._-graft-PEtOx is close to the corresponding characteristic for the previously investigated thermosensitive graft copolymers with PAlOx side chains and flexible-chain polyester backbones [69,70,71]. Thus, both copolymers under study are relatively loose brushes: in the APE_r.ch._-graft-PEtOx macromolecules, only about a half of the backbone units contain side chains, while the APE_r.ch._-graft-PiPrOx contains a quarter ones.

The difference in z determines the differences in the average distance Δ*L* = λ_0-APE_/*z* along the chain between the adjacent grafted chains (Figure 10b) and in the number *f*_sc_ = *L*_APE_/Δ*L* = *z*·*N*_APE_ of the latter for the studied copolymers (Table 2). It is interesting to compare the ΔL values with the contour length of the side chains *L*_sc_ = λ_0-sc_·*N*_sc_, where λ_0-sc_ = 0.378 nm [84] is the projection length of the PAlOx monomer unit. As can be seen from Table 2, for the APE_r.ch._-graft-PEtOx, the length *L*_sc_ is almost 10 times greater than Δ*L*. For the APE_r.ch._-graft-PiPrOx, the difference in the compared characteristics is much less, *L*_sc_/Δ*L* ≈ 2. Consequently, in a selective solvent, the main chain in the APE_r.ch._-graft-PEtOx macromolecules is sufficiently well shielded from the solvent, while in the case of the APE_r.ch._-graft-PiPrOx, the APE_r.ch._ chain is available to solvent molecules.

As is known, PAlOx are flexible-chain polymers with a Kuhn segment length *A* = 1.7 nm. Accordingly, the side chains of the APE_r.ch._-graft-PEtOx contain about 16 Kuhn segments, that is, they are in the Gaussian region in length. The Gaussian coil can be roughly modeled by an ellipsoid of revolution, the major axis of which is *H* = 1.4<*h*^2^>^1/2^, and the minor axis is *Q* = 0.7<*h*^2^>^1/2^ (Figure 10c) [85]. For the APE_r.ch._-graft-PEtOx copolymer, we have *H* = 1.4 × (*L*_sc_*A*)^1/2^ = 1.4 × (28 × 1.7)^1/2^ = 9.7 nm and *Q* = *H*/2 = 4.8 nm, that is, both *H* and *Q* is greater than the distance between the adjacent grafted chains Δ*L*. Consequently, steric repulsion occurs between the adjacent PEtOx chains, and their curvature decreases. It can be assumed that the PEtOx coil is “stretched” along the long axis, and the value of H increases. The interaction of the side chains in molecular brushes leads to the straightening of the main chain; however, if the main chain is a rigid-chain polymer, this effect is insignificant [11]. Then, the APE_r.ch._-graft-PEtOx macromolecule can be modeled with a curved thick spherocylinder (Figure 10d). Its radius *R*_sph_ is knowingly less than the length of the PEtOx side chains and greater than *H*, that is, 10 nm < *R*_sph_ < 28 nm. The length of the axis of the curved spherocylinder *L*_sph_ is the sum of the contour length of the macroinitiator and the contribution of two side chains, that is, *L*_APE_ = 71 nm < *L*_sph_ < *L*_APE_ + 2*H* = 90 nm.

In the APE_r.ch._-graft-PiPrOx copolymer, the side chains contain only six to seven Kuhn segments, and their conformation should be described within the framework of the Porod model. Then, in accordance with the ratio (5), the rms distance <*h*^2^>^1/2^ between the ends of the PiPrOx chain is about 5 nm. Hence, the side chains of the APE_r.ch._-graft-PiPrOx are markedly curled up. Since <*h*^2^>^1/2^ and Δ*L* are comparable, steric interactions between PiPrOx chains are weak, and it can be assumed that the APE_r.ch_. chain does not change the conformation when passing from the macroinitiator to the graft copolymer. The APE_r.ch._-graft-PiPrOx macromolecules can also be modeled by a worm-shaped spherocylinder. Its radius is small, less than 5 nm (that is, <*h*^2^>^1/2^ for the side PiPrOx chains), and the axis length is close to 80 nm ≈ *L*_APE_ + 2<*h*^2^>^1/2^ (Figure 10e). Probably, it is the difference in the thickness of the graft copolymer molecules that determines the difference in the values of the hydrodynamic radius for the APE_r.ch._-graft-PEtOx and the APE_r.ch._-graft-PiPrOx (Table 1).

### 2.3. Self-Organization in Aqueous Solutions of the APE_r.ch._-graft-PEtOx and APE_r.ch._-graft-PiPrOx Grafted Copolymers

**Figure 11:**
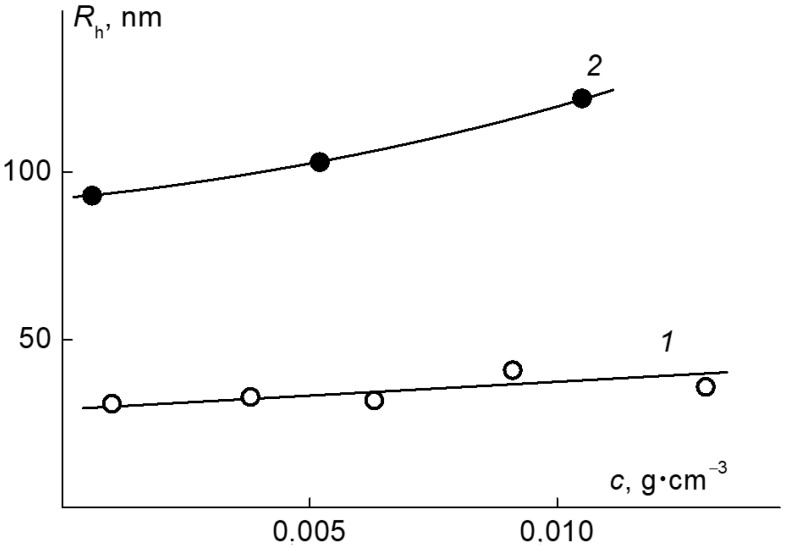
The dependence of the hydrodynamic radii of the dissolved species on the concentration in the APE_r.ch._-graft-PEtOx solutions at 21 °C (1) and the APE_r.ch._-graft-PiPrOx at 10 °C (2).

In the studied range of concentrations at a temperature T < 30 °C, aqueous solutions of the APE_r.ch._-graft-PEtOx were molecularly dispersed. As can be seen in Figure 11, there is a tendency towards a decrease in the hydrodynamic radius *R*_h_ with dilution. However, this change is small, and it fits within the experimental error.

**Figure 12:**
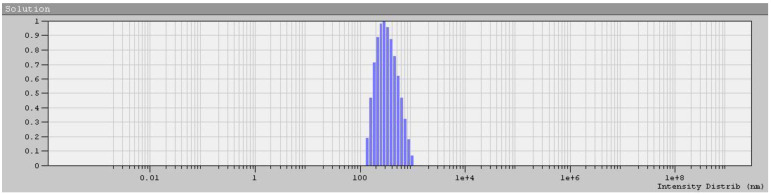
*R*_h_ spectrum obtained by DLS for the APE_r.ch._-graft-PiPrOx aqueous solution at c = 1.05 × 10^−2^ g/cm^3^ and T = 10 °C.

**Figure 13:**
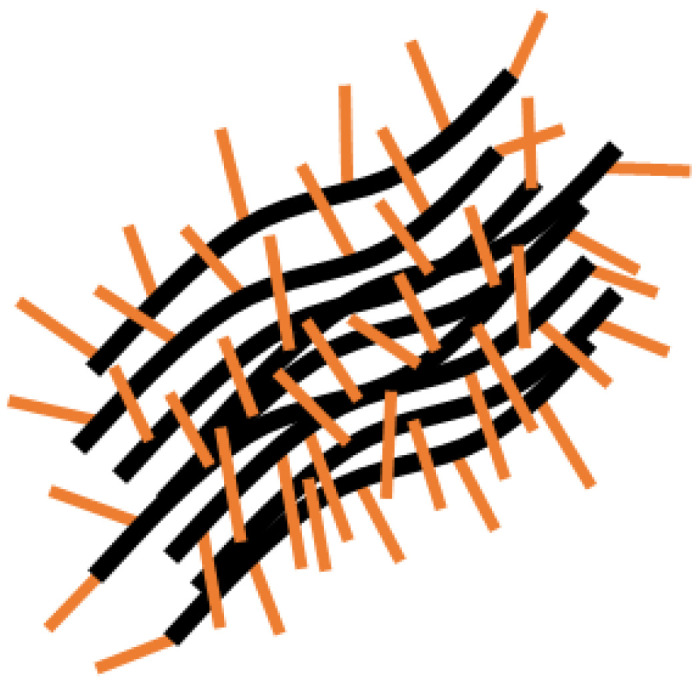
Aggregation of the APE_r.ch._-graft-PiPrOx macromolecules in water.

Aqueous solutions of the APE_r.ch._-graft-PiPrOx at 21 °C were slightly cloudy. Even at the lowest temperatures of the experiment (7 °C), they were opalescent. However, hydrodynamic size distributions obtained for them by DLS were unimodal (Figure 12). The hydrodynamic size *R*_h_ of scattering species was almost an order of magnitude higher than the *R*_h_ value obtained in chloroform (Table 1), which indicates the aggregation of the APE_r.ch._-graft-PiPrOx macromolecules in water. Aggregation is caused by hydrophobic interactions of the APE_r.ch._ backbones, which are poorly screened from the solvent, since the grafting density of water-soluble PiPrOx chains is low (*z* = 0.26), and these chains themselves are short (*L*_sc_/Δ*L* ≈ 2). As demonstrated by the example of molecular brushes with a flexible polyester backbone with short PiPrOx side chains, the mechanism of hydrophobic interactions is intramolecular: the backbone collapses, forming a core shielded from the solvent by a hydrophilic PiPrOx corona [71]. Unlike a brush with a flexible chain backbone, the rigid backbones of APE_r.ch._-graft-PiPrOx cannot fold, and it can be assumed that in order to form a solubilizing shell consisting of hydrophilic short side chains, the PiPrOx chains must be packed tightly to each other, forming bundles or sheaves (Figure 13).

**Figure 14:**
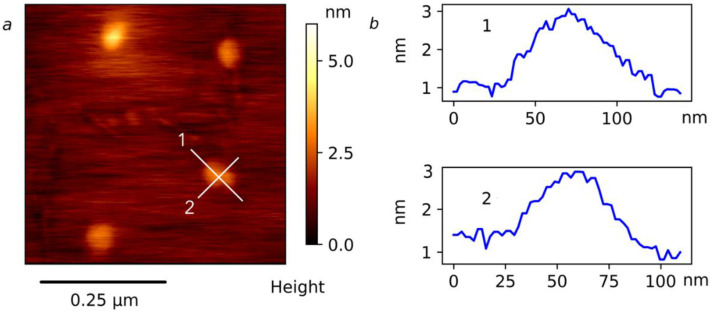
AFM topography image for mica surface with deposited APE_r.ch._-graft-PiPrOx (**a**) and the profiles (**b**) corresponding to the white lines in (**a**).

This aggregation model is supported by the AFM experiment. The AFM measurements for the APE_r.ch._-graft-PiPrOx sample have shown approximately uniform ellipsoidal-shaped particles distributed along the surface of mica (Figure 14a). Cross-sections measured on the individual nanostructures are given in Figure 14b and Figure S2. This allows an estimation of the dimensions of ellipsoids as 43–56 nm and 64–76 nm in width and length, respectively.

The hydrodynamic radius of the discussed particles *R*_h_ increased from 90 to 120 nm (Figure 11) as the concentration increased, which is in agreement with the AFM data. Augmentation of the hydrodynamic radii of aggregates with the concentration was observed before for molecular brushes with a flexible aromatic polyester backbone and PEtOx [70] and PiPrOx [71] side chains. The increase is caused by an enhancement of the hydrophobic interactions between the hydrophobic main chains of the grafted copolymers, especially between unsubstituted monomer units of the main chains, where they are less screened by hydrophilic side chains. 

**Figure 15:**
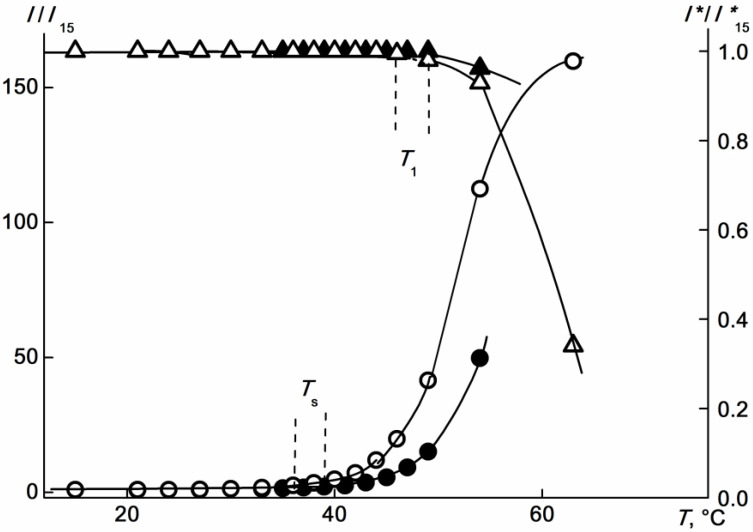
Temperature dependences of *I*/*I*_15_ (circles) and I*/I*_15_ (triangles) for the APE_r.ch._-graft-PEtOx solutions at c = 0.0063 (open symbols) and 0.0038 g·cm^−3^ (black symbols). *I*_15_ and I*_15_ are the light scattering intensity and the optical transmission at 15 °C, respectively.

The temperature dependences of the scattered light intensity *I* and optical transmission I* for the APE_r.ch._-graft-PEtOx are shown in Figure 15. Similar plots of *I*/*I*_15_(T) and I*/I*_15_(*T*) and *I*_15_ and I*_15_, being the light scattering intensity and optical transmission at 15 °C, respectively, were obtained for other concentrations. The temperature of the onset of phase separation *T*_1_ was determined as the temperature corresponding to the onset of a decrease in I*. Note that the termination of phase separation in the range of accessible temperatures was not observed at any of the studied concentrations, and for none of the studied APE_r.ch._-graft-PEtOx solutions was it possible to achieve zero optical transmission. Unlike I*, the value of which was constant up to *T*_1_, the intensity of the scattered light began to change at a temperature *T*_s_, that is, long before the start of phase separation (Figure 15). The change in *I* was smooth, its rate increased with temperature, at least up to the temperature *T*_1_. Similar *I*(*T*) dependences were previously observed for thermosensitive polymer brushes, in particular, for graft copolymers with flexible-chain polyester backbones and side chains of poly-2-ethyl-2-oxazoline [69].

**Figure 16:**
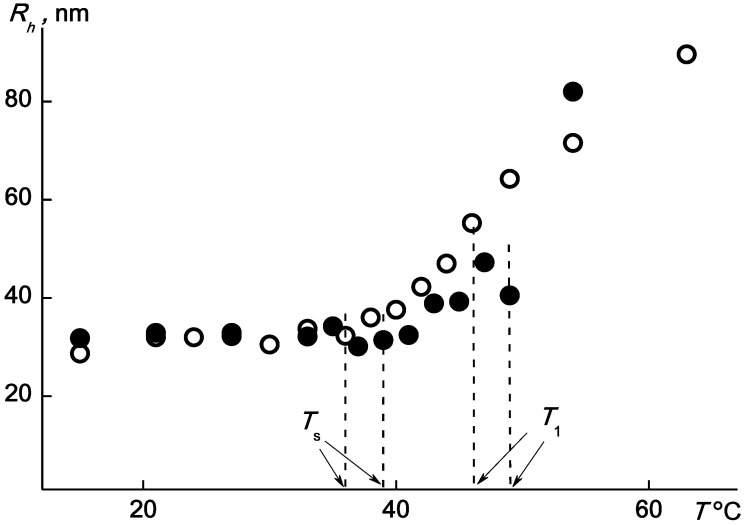
The temperature dependences of *R*_h_ for the APE_r.ch._-graft-PEtOx solutions at c = 0.0063 (open symbols) and 0.0038 g·cm^−3^ (black symbols).

The observed change in the scattered light intensity is due to the aggregation of the APE_r.ch._-graft-PEtOx macromolecules. As can be seen in Figure 16, at about the temperature *T*_s_, the value of the hydrodynamic radius *R*_h_ of the scattering species begins to increase. This reflects aggregation due to dehydration of PEtOx units and, accordingly, a decrease in the solubility of graft copolymers with increasing *T*. Near *T*_1_, the rate of change in *R*_h_ increases. The maximum values of the hydrodynamic radii of the aggregates reach 100 nm.

**Figure 17:**
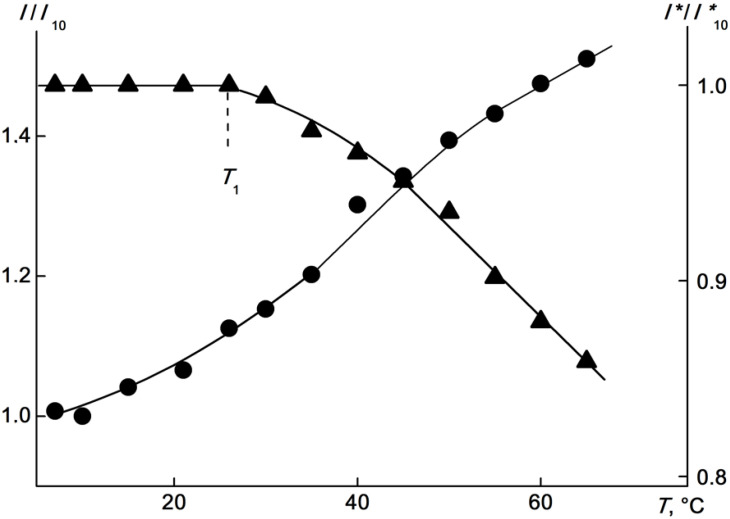
The temperature dependencies of *I*/*I*_10_ (circles) and I*/I*_10_ (triangles) for the APE_r.ch._-graft-PiPrOx solutions at c = 0.0052 g·cm^−3^. *I*_10_ and I*_10_ are the light scattering intensity and the optical transmission at 10 °C, respectively.

**Figure 18:**
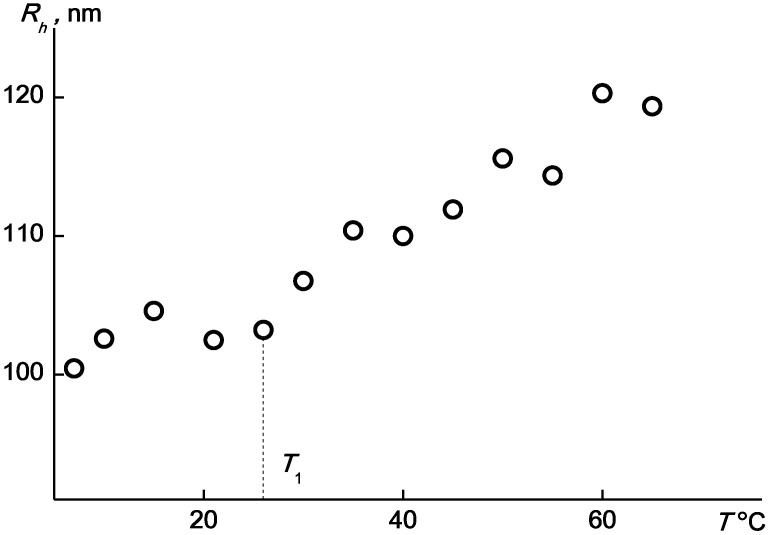
The temperature dependence of *R*_h_ for the APE_r.ch._-graft-PiPrOx solutions at c = 0.0052 g·cm^−3^.

The temperature dependences of *I*, I*, and *R*_h_ for solutions of the graft copolymer with PiPrOx side chains (Figure 17 and Figure 18) are qualitatively similar to that observed for the APE_r.ch._-graft-PEtOx. On the other hand, the changes in *I*, I*, and *R*_h_ described above for the APE_r.ch._-graft-PEtOx, in the case of APE_r.ch._-graft-PiPrOx, occur at lower temperatures. This behavior is due to the higher hydrophobicity of the APE_r.ch._-graft-PiPrOx. Indeed, the fraction ω of hydrophobic fragments in the APE_r.ch._-graft-PiPrOx macromolecules is about 50 mol%, while for the APE_r.ch._-graft-PEtOx, ω = 13 mol% (Table 3). In addition, as mentioned above, the hydrophobic backbone in the APE_r.ch._-graft-PiPrOx is much more accessible to the solvent due to the low grafting density *z* of the side chains and the relatively short length *L*_sc_ of the latter. Therefore, aqueous solutions of the APE_r.ch._-graft-PiPrOx are not molecular, resulting in high *R*_h_ values at low *T*. Heating them leads to a further increase in hydrophobicity due to the dehydration of PiPrOx units and, accordingly, to an increase in the size of aggregates (Figure 18), which causes an increase in *I* (Figure 17). Note that in the studied concentration range, the change in I and *R*_h_ begins at a very low temperature *T* = 7 °C (Figure 17 and Figure 18). In contrast to the APE_r.ch._-graft-PEtOx solutions, the dependences of I and *R*_h_ for the APE_r.ch._-graft-PiPrOx are more monotonic; they do not show an increase in the rate of *I* and *R*_h_ change at a temperature around *T*_1_.

**Figure 19:**
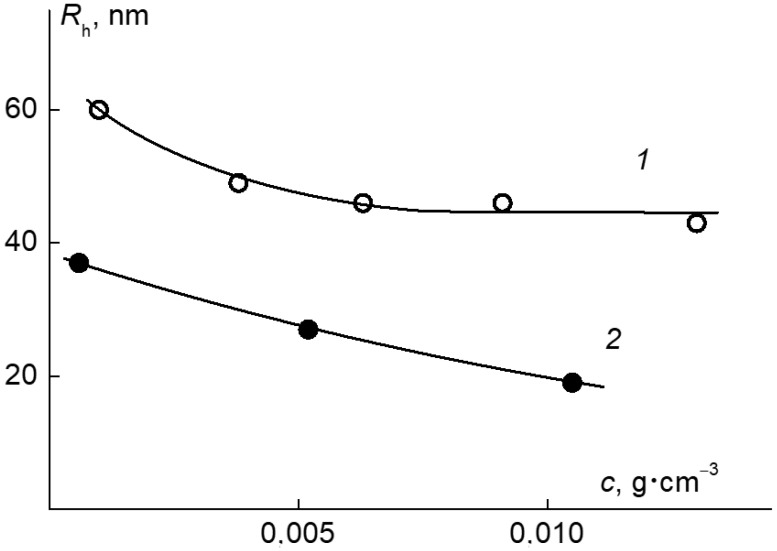
The concentration dependences of the phase separation temperatures of the APE_r.ch._-*graft*-PEtOx (1) and APE_r.ch._-graft-PiPrOx (2) solutions.

Figure 19 shows the concentration dependences of the phase separation temperatures *T*_1_. For both studied copolymers, the *T*_1_ values decrease with increasing c, which is typical for dilute solutions of thermosensitive polymers. For the APE_r.ch._-graft-PEtOx, the *T*_1_(c) dependence flattens out in the region c > 0.0063 g·cm^−3^, which makes it possible to reliably determine the LCST. For the APE_r.ch._-graft-PiPrOx solutions, the temperature *T*_1_ depends on the concentration over the entire studied range of c. Accordingly, it can be argued that for this graft copolymer, LCST is noticeably lower than the value *T*_1_ = 20 °C for the solution with the highest concentration. 

The LCST = 45 °C for the APE_r.ch._-graft-PEtOx copolymer with PEtOx chains is slightly lower than the LCST for linear PEtOx [66,86,87], which may be due to both the influence of the architecture and hydrophobicity of the APE_r.ch._-graft-PEtOx macromolecules, and the molar mass, since its total value for PEtOx chains in the polymer brush is higher than the typical molar mass values for linear PEtOx. A similar situation takes place for the APE_r.ch._-graft-PiPrOx, but in this case, the difference in LCST for the graft copolymer and the linear polymer is slightly larger [87,88,89], which can be explained by the large fraction of hydrophobic fragments.

It seems interesting to compare the obtained data with the LCST for graft copolymers with PEtOx and PiPrOx side chains and flexible polyester main chains (Table 3). For the copolymers APE_6_-graft-PEtOx with a spacer –(CH2)_6_– in the main chain, LCST is 6 and 11 °C lower than the LCST for the APE_r.ch._-graft-PEtOx [70]. This difference cannot be explained by the difference in the molar fraction of hydrophobic fragments, since the ω values are higher for the APE_6_-graft-PEtOx. The relative length *L*_sc_ of the side chains, more precisely the ratio *L*_sc_/Δ*L* of this length *L*_sc_ to the distance between two adjacent side chains Δ*L*, for copolymers with a flexible main chain is 4–6 times less (Table 3). Therefore, in the APE_r.ch._-graft-PEtOx, the APE_r.ch._ chain should be better shielded than the main chain in the APE_6_-graft-PEtOx. However, in reality, this is not the case, since the flexible backbone of the APE_6_-graft-PEtOx collapses, sharply decreasing the distance Δ*L* and increasing the density of the PEtOx corona. In addition, the molar mass can contribute to the decrease in the LCST upon passing from the APE_6_-graft-PEtOx to the APE_r.ch._-graft-PEtOx, which differs for the compared graft copolymers by 2.8 and 3.5 times.

For the copolymers with PiPrOx side chains, the MM and *L*_sc_/Δ*L* ratio are practically the same, and the decrease in the LCST for the APE_r.ch._-graft-PiPrOx as compared to the APE_8_-graft-PEtOx with a flexible spacer –(CH_2_)_8_– in the main chain [71] is probably primarily due to the greater hydrophobicity of the APE_r.ch._-graft-PiPrOx macromolecules. Poor protection of the APE_r.ch._ backbones results in their hydrophobic interactions, to which, upon heating, interactions of dehydrated PiPrOx units are added.

### 2.4. Kinetics of Aggregation in Aqueous Solutions of the APE_r.ch._-graft-PEtOx and the APE_r.ch._-graft-PiPrOx

All the results discussed above refer to the “equilibrium” state of solutions, that is, conditions when their characteristics are constant over time. The times *t*_eq_ and t*_eq_ for the APE_r.ch._-graft-PEtOx and APE_r.ch._-graft-PiPrOx solutions to reach the equilibrium state after the temperature change were found by flattening of the *I*(*T*) or I*(*T*) dependences, which are shown in Figure S3.

**Figure 20:**
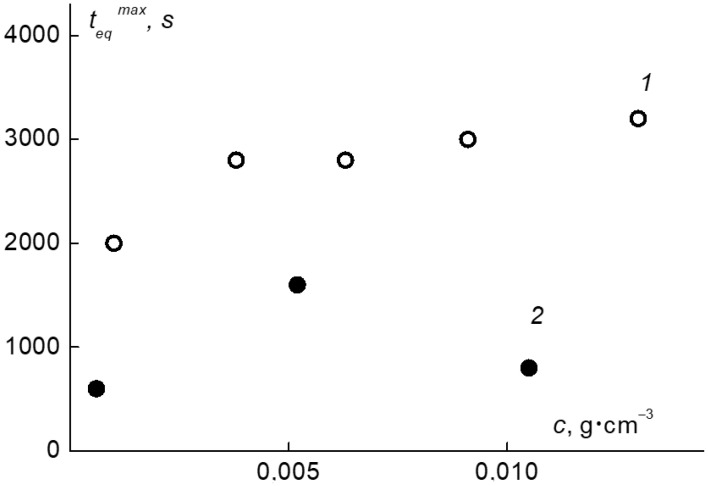
The concentration dependence of *t*_eq_^max^ for the APE_r.ch._-graft-PEtOx (1) and APE_r.ch._-graft-PiPrOx (2) solutions.

For all solutions, *t*_eq_ values depended on *T*. For the APE_r.ch._-graft-PEtOx, they were minimal at low temperatures, increased with heating, taking the maximum value *t*_eq_^max^ near the *T*_1_ for a given concentration, and then *t*_eq_ decreased (Figure S4). Note that similar dependences were previously observed for thermosensitive star-shaped poly-2-alkyl-2-oxazolines and PAlOx graft copolymers [70,71,90]. As seen in Figure 20, for the APE_r.ch._-graft-PEtOx solutions, *t*_eq_^max^ decreases with dilution. The most important thing is that for both polymers, the obtained values are noticeably lower than *t*_eq_^max^ determined earlier for solutions of the APE_6_-graft-PEtOx and APE_8_-graft-PiPrOx graft copolymers. In particular, for the APE_6_-graft-PEtOx, *t*_eq_^max^ reached 12,000 s [70]. The acceleration of self-organization processes may be due to the fact that the APE_r.ch._-graft-PEtOx sample proceeds mainly by the aggregation mechanism, while in the APE_6_-graft-PEtOx solutions, the main chain is also compacted, which leads to an increase in the density of the hydrophilic corona and hinders the contacts of hydrophobic nuclei [69,70]. 

For the APE_r.ch._-graft-PiPrOx solutions, the “settling” times have the smallest values: at low temperatures, the *t*_eq_ is in the range from 200 to 400 s. Such values are typical for linear thermoresponsive polymers [91,92,93,94]. The maximum times *t*_eq_^max^ do not exceed 2000 s, which is 2.5–6 times less than the *t*_eq_^max^ for the APE_8_-graft-PiPrOx with a flexible main chain [71]. The high rate of aggregation in the APE_r.ch._-graft-PiPrOx solutions upon heating can be explained by the fact that its macromolecules were already aggregated before heating, and the aggregates form faster by combining ready-made supramolecular structures.

## 3. Materials and Methods

### 3.1. Synthesis

2-[4-(2-Br-ethyl)]phenylsulfonylhydroquinone (1) [73] and 2-isopropyl-2-oxazoline [95] were synthesized according to the known procedures. 1-Chloronaphthalene and 1,1,2,2-tetrachloroethane (Aldrich) as well as oxazolines were dried over calcium hydride and distilled.

NMR spectra were recorded on a Bruker AC 400 spectrometer (400 MHz) for solutions in CDCl_3_. Dialysis was performed with the use of dialysis bags (CellaSep, Orange Scientific Braine-l’Alleud, Belgium) with an MWCO of 3500 Da. 

The chromatographic analysis was performed on a Shimadzu LC-20AD chromatograph equipped with a SDA0830055E1 column (PSS SDV 50 Å (5 µm) 300 mm × 8.0 mm, Mainz, Germany) and a refractometric detector. A solution of LiBr in DMF (0.1 mol/L) at 60 °C was used as the mobile phase. Calibration was performed relative to poly(ethylene glycol) standards (*M*_w_ = 6 × 10^2^–4 × 10^4^).

#### 3.1.1. Poly(2-[4-(2-Br-ethyl)phenylsulfonyl]-1,4-phenylene-2’,5’-biphenyldicarboxylate Synthesis

A flask equipped with a stirrer and a gas-supplying tube was charged with 1 (4.23 g, 0.01 mol), 2,5-dichlorocarbonylbiphenyl (2.79 g, 0.01 mol), and 1-chloronaphthalene (30 mL). The obtained mixture was purged with dry argon and heated up to 200 °C under a flow of gas. The reaction mixture was kept at 200 °C for 2 h. The polymer was precipitated with hexane, continuously extracted with hexane in Soxlet apparatus for 6 h, and dried. Yield 6.5 g (93%).

^1^H NMR (CDCl_3_, δ ppm.): 3.22 (d, ArCH_2_CH_2_Br), 3.56 (d, ArCH_2_CH_2_Br), 7.11–8.43 (m, Ar–H)

#### 3.1.2. Polymerization of 2-Alkyl-2-Oxazolines on the Polyester Macroinitiator

A solution of initiator and monomer in 1,1,2,2-tetrachloroethane (feed ratio monomer/functional groups of macroinitiator 30/1 for 2-isopropyl-2-oxazoline and 100/1 for 2-ethyl-2-oxazoline) was heated under argon for 72 h at 70 °C. The solvent was distilled off in vacuum, the polymer was dissolved in ethanol, dialyzed against water for 48 h, and freeze-dried.

#### 3.1.3. Hydrolysis of Graft-Copolymers

A solution of 0.1 g of a graft-copolymer in 5 mL of 1 M KOH in 2-methoxyethanol was heated under reflux for 10 min, after which it was neutralized and evaporated to dryness in vacuo. The residue was dissolved in 5 mL of ethanol, dialyzed against sodium bicarbonate (concentration 0.1 mol/L) using CellaSep dialysis bags with MWCO 1000 Da, and freeze-dried. The product was dissolved in 15 mL of propionic anhydride or isobutyric anhydride, heated at 50 °C for 30 min, and evaporated under reduced pressure.

### 3.2. Determination of Molar Mass and Hydrodynamic Characteristics of Polymers

Weight-average molar masses *M*_w_ of the macroinitiator APE_r.ch._ and the APE_r.ch._-graft-PAlOx graft copolymers, and the hydrodynamic radii R_h_ of their macromolecules were determined in dilute solutions in organic solvents by static (SLS) and dynamic (DLS) light scattering. Tetrachloroethane (dynamic viscosity η_0_ = 1.74 × 10^−3^ Pa·s, density *ρ*_0_ = 1.595 g/cm^3^ and refractive index *n*_0_ = 1.49) was used as a solvent for the APE_r.ch._, and it was chloroform for the molecular brushes (η_0_ = 0.542 × 10^−3^ Pa·s, *ρ*_0_ = 1.483 g/cm^3^ and *n*_0_ = 1.4467), since associative phenomena were absent in them. 

The measurements were carried out in a Photocor Complex instrument (Photocor Instrument Inc., Russia), which was equipped with a Photocor-PC2 correlator with 288 channels, as well as a Photocor-PD detector for measuring the intensity of transmitted light. A Photocor-DL semiconductor laser with a wavelength *λ*_0_ = 659.1 nm was used as a light source. Calibration was carried out using toluene, the absolute scattering intensity of which was *R*_v_ = 1.38×10^−5^ cm^−1^. Before measurements, the solutions were filtered into dust-free cells using Chromafil polyamide filters (Macherey-Nagel GmbH & Co. KG, Dueren, Germany) with a pore size of 0.45 μm.

For all solutions, there was no scattered light asymmetry, and *M*_w_ values were obtained by the usual Debye technique by measuring the light scattering intensity at an angle of 90°. The plots for *M*_w_ determination for the macroinitiator and the grafted copolymers are shown on Figure S5. The values of *M*_w_ were calculated using the equation:

(7) $$\frac{cH}{I}=\frac{1}{M_{w}}+2A_{2}c\;,$$

where *I*_90_ is the intensity of the light scattering at the 90° angle, *A*_2_ is the second virial coefficient, *H* is the optical constant, and the value of *H* is:

(8) $$H=\frac{4\pi^{2}n_{{}_{0}}^{2}{\left(dn/dc\right)}^{2}}{N_{A}\lambda_{0}^{4}},$$

where *N*_A_ is the Avogadro’s number.

The scattered light intensity distributions were unimodal. The hydrodynamic radii of the macromolecules *R*_h_(*c*) collected at concentrations c did not depend on *c* (Figure S1), and the *R*_h_(*c*) values were averaged over the concentration to obtain the *R*_h_.

The refractive index *n* was measured on a RA-620 refractometer (KEM, Kyoto, Japan). The refractive index increment *dn*/*dc*, which is a factor in (7), was calculated as a slope of the dependence (*n* − *n*_0_)/*c* on *c*, where *n* is the refractive index of the solution taken at a concentration *c*.

### 3.3. Study of the Solutions’ Phase Separation upon Heating

The thermal sensitivity of the APE_r.ch._-graft-PAlOx copolymers in aqueous solutions was studied by light scattering and turbidimetry using the Photocor Complex device described above. The temperature T was controlled with an accuracy of 0.1 °C, changing it discretely with a step from 5.0 °C at low temperatures to 1.0 °C near the phase separation interval.

The scattered light intensity I and the transmitted light intensity I* were measured as a function of *T* with increasing temperature. The temperatures *T*_1_ of the beginning of phase separation were determined from the dependences *I*(*T*) and I*(*T*), taking as *T*_1_ the temperatures at which a decrease in I* began. The hydrodynamic radii *R*_h_ of scattering species were measured as a function of *T* The measurements of the particles’ size were carried out after reaching the equilibrium state of the solutions, that is, after the values of *I* and I* reached constant values over time.

### 3.4. Microscopic Investigation

The surface morphology of the samples was investigated by the AFM method on the SPM-9700HT scanning probe microscope equipped with the SPM software v.4.76.1 (Shimadzu, Kyoto, Japan) using NSG30-SS Silicon probes with the radius of the tip curvature of 2 nm produced by “TipsNano” (Tallinn, Estonia). To take the sample, the tapping mode measurements were conducted in air using mica as a support.

## 4. Conclusions

New diphylic grafted copolymers were synthesized successfully. A polycondensation aromatic polyester served as a macroinitiator to graft poly(2-ethyl-2-oxazoline) and poly(2-isopropyl-2-oxazoline) side chains by cationic polymerization. A fundamentally different chemical class of the backbone and the side chains ensure diphilicity of the resulting polymer brushes. Analysis of their molecular and architectural characteristics made it possible to conclude that they can be considered as loose brushes, with a grafting degree of 0.53 and 0.27 for the APE_r.ch._-graft-PEtOx and the APE_r.ch._-graft-PiPrOx, respectively. In contrast to the previously studied molecular brushes APE-graft-PAlOx, in this case, the macroinitiator was a rigid-chain polymer. The Kuhn segment length for the APE_r.ch._ macroinitiator is estimated to be of the order of 23 nm, which results in specific properties of the polymer brushes as in organics as in a selective solvent. In chloroform, the APE_r.ch._-graft-PEtOx and APE_r.ch._-graft-PiPrOx macromolecules take a wormlike cylindrical conformation, the asymmetry of which depends on the PAlOx side chains’ length.

It was shown that architectural parameters are essential for their conformational properties in selective solvents, self-organization, and thermoresponsiveness. Due to high equilibrium rigidity of the main chain, the nature of the self-assembly process of the graft copolymers of the aromatic polyester with PAlOx side chains differs significantly from that of the flexible-chain polymer brushes with similar side chains. Whereas a flexible APE main chain with short side chains is sufficiently labile and capable of changing conformation in various solvents, the rigid hydrophobic APE_r.ch._ backbone determines either the intra- or intermolecular organization of macromolecules, depending on the length of the side chains and the distance between the grafting points. A rigid-chain brush with a low side chain grafting density takes on a cylindrical wormlike conformation in the case of sufficiently long PAlOx side chains and aggregate in water in big structures if the side chains are short.

The range of thermosensitivity of the rigid-chain APE_r.ch._ copolymer, which is conditioned by the thermoresponsiveness of the PAlOx side chains, was studied. The LCST of the APE_r.ch._-graft-PEtOx under consideration, at given molecular and architectural characteristics, is assumed to be around 45 °C, whereas for the APE_r.ch._-graft-PiPrOx, the LCST is much lower than 20 °C. The phase separation temperatures are determined by both the structure and the length of the side chains, and the grafting density. Thus, architecture parameters play a prominent role as in conformational and aggregative properties as in the thermoresponsive behavior of hybrid graft copolymers with a hydrophobic aromatic polyester main chain and hydrophilic thermosensitive polyalkyloxazoline side chains.

## Figures and Tables

**Table 1:** Molecular characteristics of the APE_r.ch._, the APE_r.ch._-graft-PEtOx, and the APE_r.ch._-*graft*-PiPrOx.

Polymer	*M*_w_, g·mol^−1^	*A*_2_ × 10^4^, cm^3^mol/g^2^	*R*_h_, nm
APE_r.ch._	26,500	0.4	12
APE_r.ch._-graft-PEtOx	208,000	−0.6	31
APE_r.ch._-graft-PiPrOx	68,000	5.9	18

**Table 2:** Structural parameters of the APE_r.ch._-graft-PEtOx and APE_r.ch._-graft-PiPrOx.

Polymer	*M*_s_, g·mol^−1^	*z*	*N*_sc_	*L*_sc_, nm	Δ*L*, nm	*f*_sc_
APE_r.ch._-graft-PEtOx	7400	0.53	75	28	2.9	24
APE_r.ch._-graft-PiPrOx	3400	0.27	30	11	5.6	13

**Table 3:** Molar masses, structural parameters, and LCST for the APE_r.ch._-graft-PEtOx, APE_6_-graft-PEtOx, APE_r.ch._-graft-PiPrOx, and APE_8_-graft-PiPrOx.

Polymer	*M*_w_, g·mol^−1^	*L*_sc_/Δ*L*	ω, mol%	LCST, °C	Reference
APE_r.ch._-graft-PEtOx	208,000	10	13	45	this work
APE_6_-graft-PEtOx ^1^	59,000	1.7	33	50	[70]
APE_6_-graft-PEtOx ^1^	75,000	2.3	24	55	[70]
APE_r.ch._-graft-PiPrOx	68,000	2.0	50	<20	this work
APE_8_-graft-PiPrOx ^2^	74,000	2.0	26	20	[71]

^1^ APE_6_-graft-PEtOx is a graft copolymer containing –(CH)_6_– spacer in the APE_6_ main chain [70]. ^2^ APE_8_-graft-PEtOx is a graft copolymer containing –(CH)_8_– spacer in the APE_8_ main chain [71].

## Data Availability

The data presented in this study are available on request from the corresponding author.

## Supplementary Materials

The following are available online at https://www.mdpi.com/article/10.3390/ijms222212265/s1.
