# Supplementary material for: Thermoresponsive Molecular Brushes with a Rigid-Chain Aromatic Polyester Backbone and Poly-2-alkyl-2-oxazoline Side Chains
Source: Int J Mol Sci. 2021 Nov 12;22(22):12265. doi: 10.3390/ijms222212265 (PMC8622345; doi:10.3390/ijms222212265)
Supplement: Supplementary file 1 [file ijms-22-12265-s001.zip › ijms-1459414-supplementary.pdf]

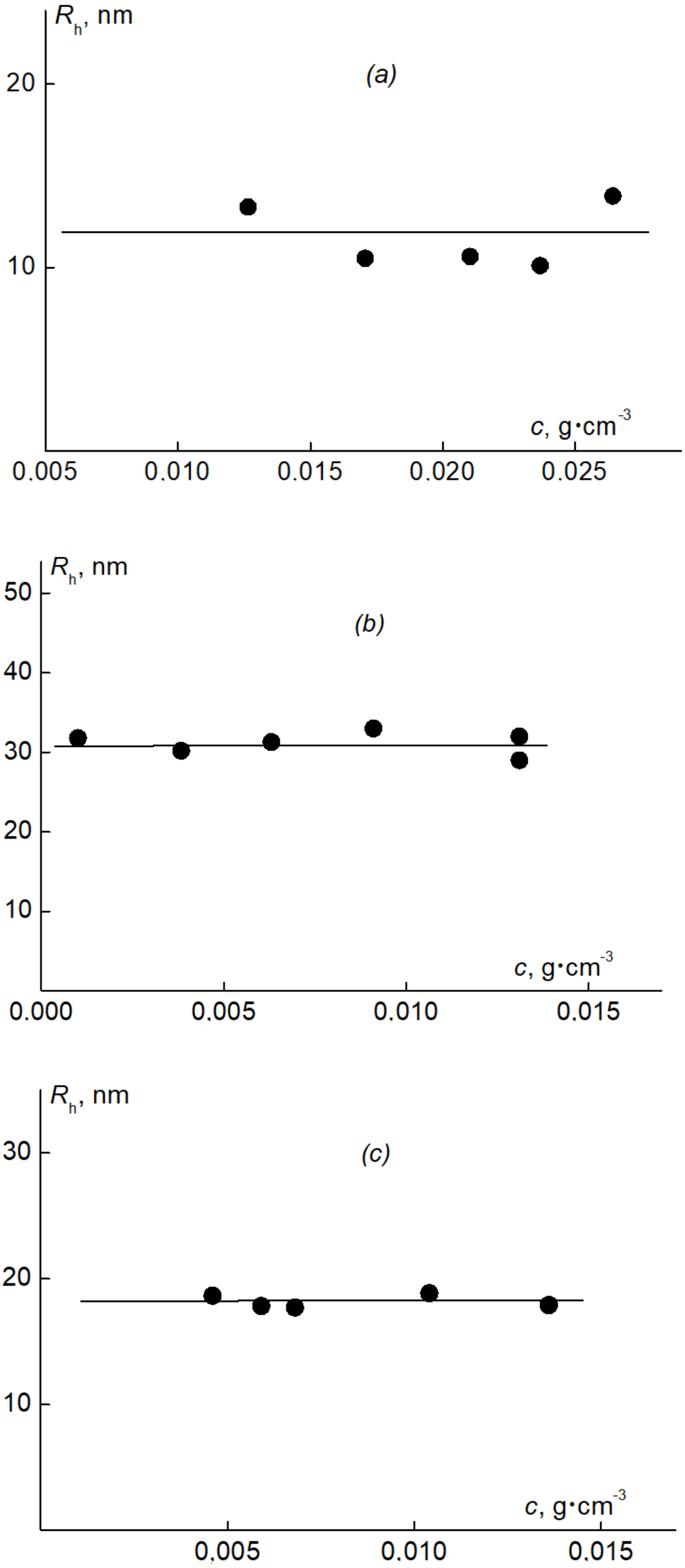

Figure S1. The concentration dependence of hydrodynamic radii plots for the APE$_{r.ch.}$ (**a**) in TCE, and the APE$_{r.ch.}$-graft-PEtOx (**b**) and the APE$_{r.ch.}$-graft-PiPrOx (**c**) in chloroform solutions.

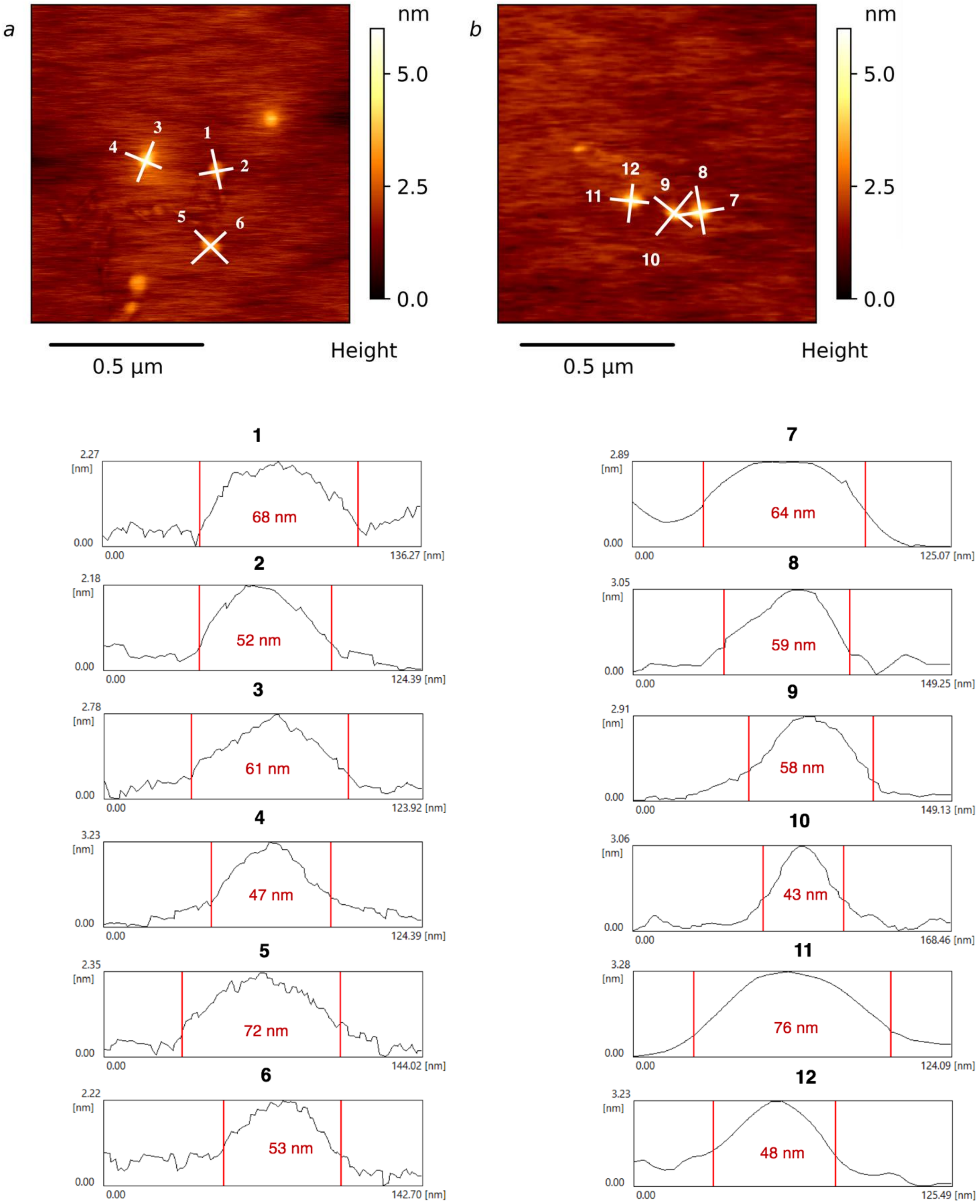

Figure S2. AFM topography images for different areas of mica surface with deposited $APE_{r.ch.}$-graft-PiPrOx (**a**,**b**) and the AFM profiles corresponding to the white lines in a and b.

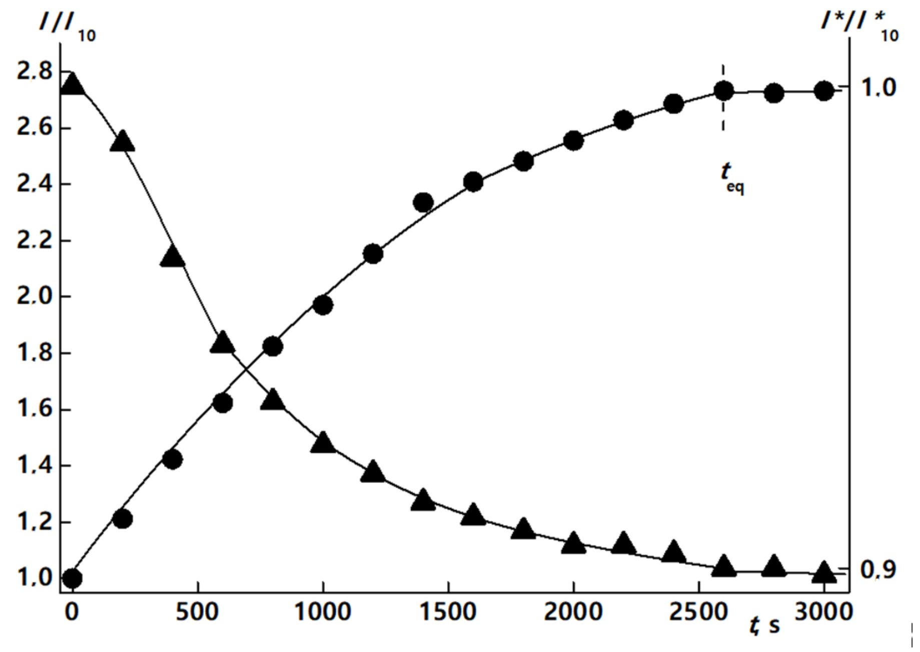

Figure S3. The progress of I/I_0 (circles) and I^*/I^*_0 (triangles) on time t for the APE$_{r.ch.}$-graft-PEtOx at c = 0.013 × g·cm^{-3} and T = 37 °C. I_0 and I^*_0 are the values of the intensity of light scattering and optical transmission at t = 0, respectively. For the time t = 0, the moment was taken when the temperature reached the set value.

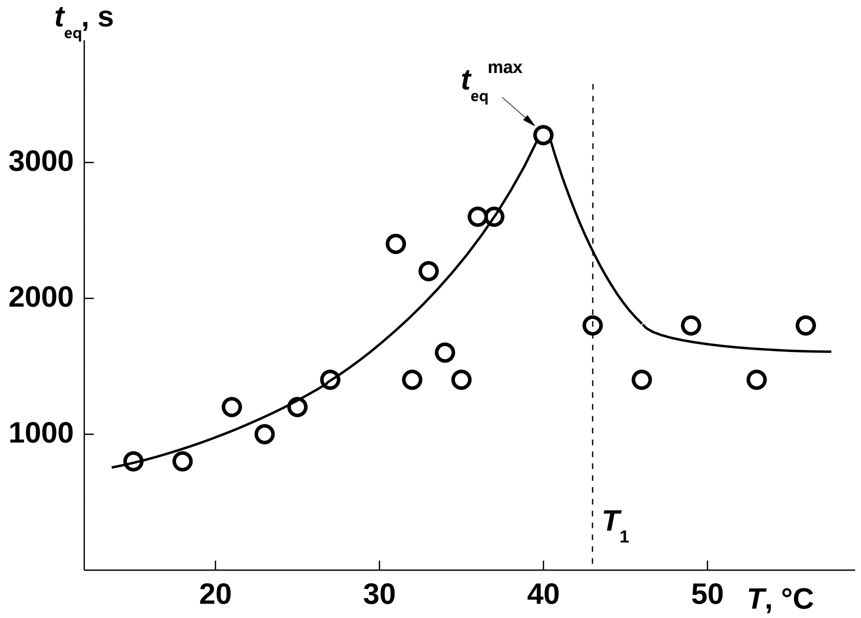

Figure S4. The dependence of t_{eq} on T for the APE$_{r.ch.}$-graft-PEtOx solution at c = 0.013 × g/cm^3.

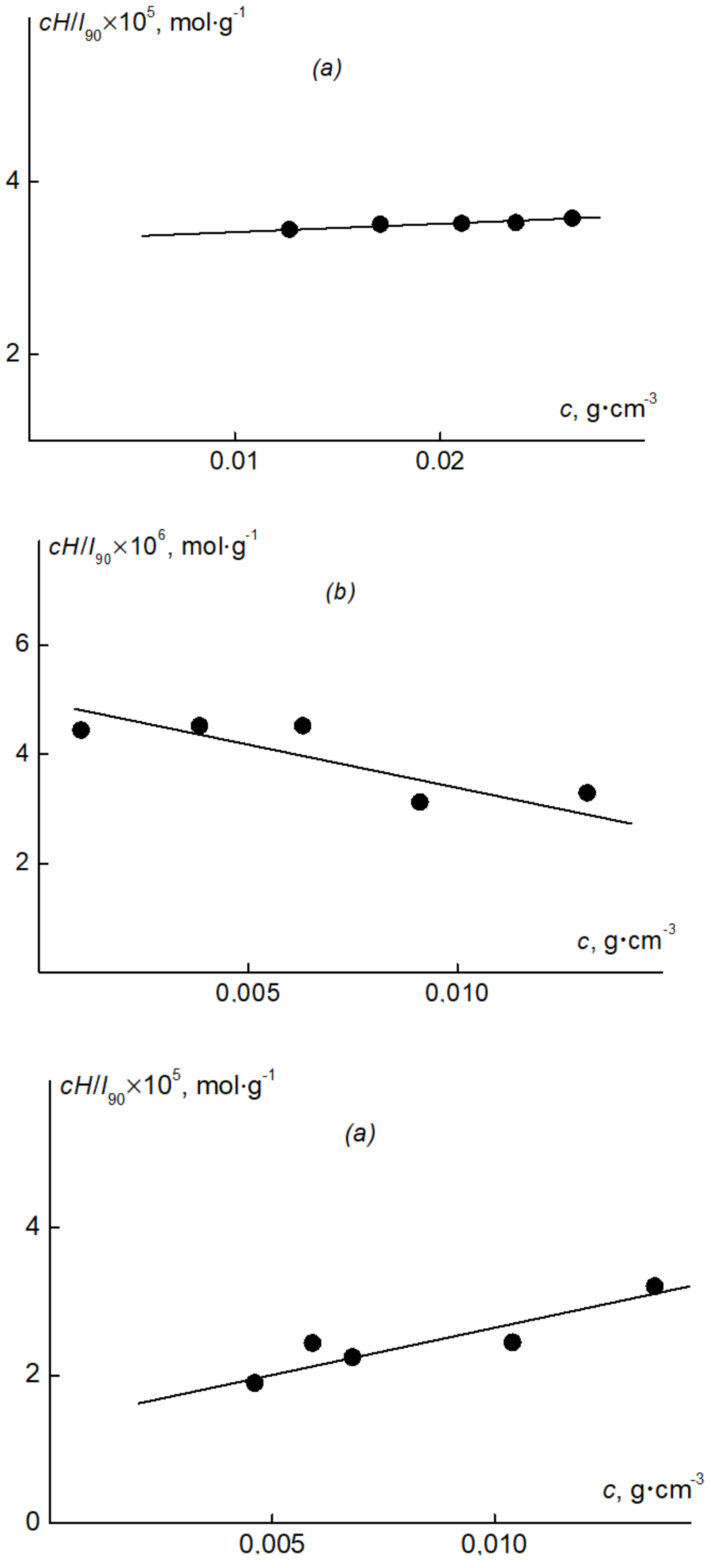

Figure S5. Debye plots for $APE_{r.ch.}$ (**a**), $APE_{r.ch.}$-graft-PEtOx (**b**), and $APE_{r.ch.}$-graft-PiPrOx (**c**).
